# Rapid speciation in the holopelagic ctenophore *Mnemiopsis* following glacial recession

**DOI:** 10.1101/2024.10.10.617593

**Published:** 2024-11-09

**Authors:** Remi N. Ketchum, Edward G. Smith, Leandra M. Toledo, Whitney B. Leach, Natalia Padillo-Anthemides, Andreas D. Baxevanis, Adam M. Reitzel, Joseph F. Ryan

**Affiliations:** 1 Whitney Laboratory for Marine Bioscience, University of Florida, St. Augustine, Florida, USA; 2 Department of Genetics, University of North Carolina at Chapel Hill, Chapel Hill, North Carolina, USA; 3 School of Life Sciences, The University of Warwick, Coventry, UK; 4 Department of Biological Sciences, Lehigh University, Bethlehem, Pennsylvania, USA; 5 Division of Intramural Research, National Human Genome Research Institute, National Institutes of Health, Bethesda, Maryland, USA; 6 Department of Biological Sciences, University of North Carolina at Charlotte, Charlotte, North Carolina, USA

## Abstract

Understanding how populations diverge is one of the oldest and most compelling questions in evolutionary biology. An in depth understanding of how this process operates in planktonic marine animals, where barriers for gene flow are seemingly absent, is critical to understanding the past, present, and future of ocean life. *Mnemiopsis* plays an important ecological role in its native habitat along the Atlantic coast of the Americas and is highly destructive in its non-native habitats in European waters. Although historical literature described three species of *Mnemiopsis*, the lack of stable morphological characters has led to the collapse of this group into a single species, *Mnemiopsis leidyi*. We generate high-quality reference genomes and use a whole-genome sequencing approach to reveal that there are two species of *Mnemiopsis* along its native range and show that historical divergence between the two species coincides with historical glacial melting. We define a hybridization zone between species and highlight that environmental sensing genes likely contribute to the invasive success of *Mnemiopsis*. Overall, this study provides insights into the fundamental question of how holopelagic species arise without clear barriers to gene flow and sheds light on the genomic mechanisms important for invasion success in a highly invasive species.

## Introduction

Speciation and species delineation is of fundamental importance to evolutionary biology, yet the vast majority of speciation literature is based on terrestrial species with small population sizes, clear physical barriers to gene flow, and external fertilization (1–3). Additionally, what we know about speciation in the ocean is largely dominated by active swimmers with well-developed dispersal abilities (i.e., fish) and coastal epibenthic fauna that are relatively sedentary in their adult stages (4). To better understand mechanisms driving speciation in the oceans, it is critical to expand this set of organisms to include animals that employ a wide range of life history strategies and inhabit other distinct marine ecosystems. Holopelagic invertebrate species spend their entire life in the water column and represent a common suite of life history traits in the ocean with high fecundities (5), large population sizes (6), and high dispersal potential.

*Mnemiopsis* (A. Agassiz, 1865) is a holopelagic genus within the phylum Ctenophora with a native range extending continuously along more than 25,000 kilometers of the Atlantic coast of the Americas from Kittery, Maine to the Valdes Peninsula in Argentina (7–9). These comb jellies are considered one of the 100 most invasive species in the world (10) and their introductions into European waters have triggered community trophic cascades and collapse of fisheries (11). The extensive geographic distribution and invasive abilities of *Mnemiopsis* are driven in part by its broad environmental tolerance (12), flexible planktivorous diet (13), extensive regeneration abilities (14), high fertility levels (15), and a capacity for self-fertilization (16). Risk management for invasive species requires genetic information to establish management units and accurate taxonomic classification is a fundamental component of effective control measures.

Despite its rich 100-year history (17) as an evo devo model and the availability of extensive genomics resources (18–21), our understanding of species boundaries in *Mnemiopsis* is subject to debate (22–26). Within the World Register of Marine Species (WoRMS), *Mnemiopsis* is currently divided into two accepted species, *Mnemiopsis leidyi* (A. Agassiz, 1865; type location Massachusetts) and *Mnemiopsis gardeni* (L. Agassiz, 1860; type location South Carolina). A third species, *Mnemiopsis mccradyi* (Mayer, 1900; type location South Carolina) is recognized as a synonym for *Mnemiopsis leidyi* (27). However, there is much confusion as to the ranges and differences in morphology between these two named species and most modern literature considers all ctenophores along the Atlantic and Gulf coasts of the United States as a single species: *Mnemiopsis leidyi* (28). Although there have been previous studies that suggest the possibility of multiple *Mnemiopsis* species (22–26), the molecular data from these studies are based on a limited set of markers and/or limited population sampling and have been insufficient to resolve this question. However, the rapid expansion of next-generation sequencing (NGS) technologies has revolutionized the study of genomic variation within and between species and presents us with a unique opportunity to test for multiple species, revisit the naming system, and explore the evolutionary changes that contribute to invasion success of holopelagic species.

We conducted whole-genome short-read sequencing of 118 individuals from 13 populations within *Mnemiopsis’s* native range along the United States Atlantic coast and generated two near chromosome-level genome assemblies of one individual from Florida and one individual from Massachusetts. We used a suite of genomic approaches to understand holopelagic population dynamics, demographic histories, changes in genomic architecture, and evolutionary adaptations. Taken together, these findings highlight barriers to gene flow in holopelagic species, mechanisms underpinning invasion success, and the importance of high spatial resolution sampling in identifying units for invasive species management.

## Results and Discussion

### Whole-genome sequencing of *Mnemiopsis* populations

**Figure 1.**
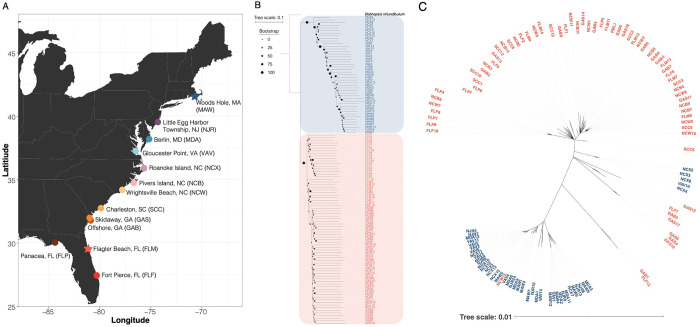
**A)** Sampling map for *Mnemiopsis* collections. Each site is colored according to the MDS plot color scheme with the site abbreviations in parentheses. Stars indicate populations that were sampled for PacBio genome sequencing and assembly. **B)** Phylogenetic tree based on nuclear SNPs, estimated by ngsDist and FastMe using 1,302 SNPs and rooted with *Bolinopsis infundibulum.* The size of the circle on the branch represents the bootstrap support. The northern and southern lineages both receive 100% bootstrap support. The colors of the sample names indicate which lineage the sample belongs to (blue; northern, red; southern). **C)** Mitochondrial genome tree generated using an alignment of the whole mitogenome and IQ-TREE v2.3.2. The colors of the sample names indicate which lineage the sample belongs to.

We sequenced the genomes of 118 *Mnemiopsis* individuals from 13 populations along the US Atlantic coast (Figure 1A, Supplementary Table 1). We generated a total of 730 Gb of Illumina reads and an average genome depth of 18.89x. From this, we generated three datasets that were appropriate for different types of analyses: the All-Sites dataset with 81,758,954 single nucleotide polymorphisms (SNPs), the Population-specific dataset with 3,856,382 SNPs, and an LD-Filtered dataset with 89,750 SNPs.

### Population genomic evidence of two *Mnemiopsis* species

We initially performed phylogenetic analyses using nuclear and mitochondrial markers from our *Mnemiopsis* samples collected along the Atlantic Coast. Our nuclear phylogenetic tree (Figure 1B) revealed the presence of two distinct clades that we will herein refer to as the northern lineage (Roanoke Island, NC to Woods Hole, MA) and the southern lineage (Pivers Island, NC to Panacea, FL). For the most part, these two distinct clades are also observed in our mitochondrial phylogenetic tree (Figure 1C). However, we identified six individuals from populations near the geographic interface of these two clades that exhibit mitonuclear discordance. Specifically, we observed one individual collected at Gloucester Point, Virginia (VAV10) and four individuals collected in Roanoke Island, North Carolina (NCX3–5,8) with northern nuclear and southern mitochondrial genotypes (Figure 1B–C). Likewise, we observed one individual collected from Wrightsville Beach, North Carolina (NCW15) with southern nuclear and northern mitochondrial genotypes. This level of mitochondrial discordance could be consistent with geographically localized hybridization and adaptive introgression, secondary contact, or demographic disparities such as sex-biased dispersal (29, 30).

**Figure 2.**
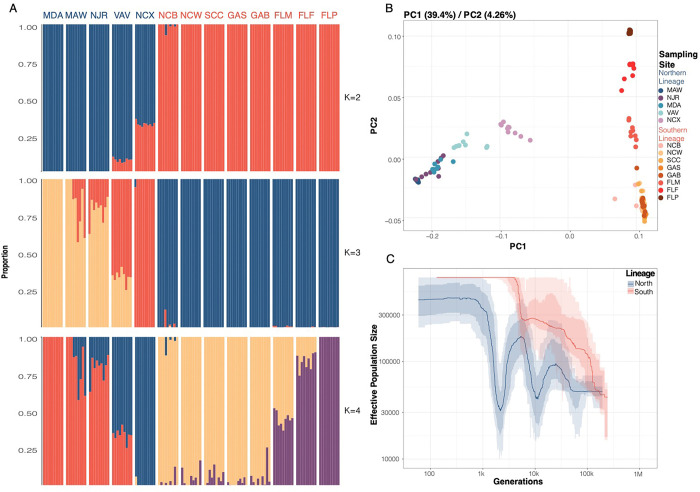
**A)** Admixture plots for the 118 *Mnemiopsis* samples collected for K=2–4 where each bar represents a single individual, and the colors represent each of the K components. **B)** MDS plot for PC1 and PC2 with the percent of total variance explained by each component shown above the plot in parentheses. Samples are colored according to their sampling location with northern lineage samples clustering on the left in blues and purples and southern lineage samples clustering on the right in reds. **C)** Stairway plot results show different demographic histories of the two lineages with generation time of one year and a mutation rate of 6.85 × 10^−8^ per base pair per year.

To further investigate these patterns, we performed admixture, multidimensional scaling (MDS), and population genetic analyses. We evaluated the presence of 2–7 populations (by adjusting the K parameter) within our dataset using ngsADMIX (31). Setting K=2 separates individuals into northern and southern lineages with the Roanoke Island, NC (NCX) and Gloucester Point, VA (VAV) samples showing signs of admixture (Figure 2A). When K=3, the Roanoke Island samples form their own subpopulation and at higher K values, more substructure within the two lineages becomes evident. MDS plots (Figure 2B) support our ngsADMIX results with the first principal component mirroring the patterns observed in K=2 and accounting for 39.4% of the variance in the data. Our observations of distinct lineages can also be found at the population level, with pairwise F_ST_ comparisons revealing that the average F_ST_ between the northern and southern lineage was 0.278 when the putative hybrid population (Roanoke Island) was included in the northern lineage and 0.315 when it was removed (Table 1). Due to these cumulative results, we also tested for evidence of hybridization in our data using *f*_3_ statistics (32). We compared Roanoke Island, NC (NCX) to the northern and southern lineage and notably, this resulted in a negative *f*3 statistic and an absolute Z score >= 3 (threshold used for significance) highlighting that Roanoke Island, NC is a hybrid population (Supplementary Table 2). A recent study (26) detected evidence of hybridization in two populations in Virginia: Gloucester Point and Wachapreague. Because we also sampled from Gloucester Point, we tested for evidence of hybridization in this population by comparing it to the northern and southern lineages (Supplementary Table 3) and did not detect evidence for hybridization. This can likely be explained by the increased statistical power of using 4.8 million SNPs versus the 96 SNPs used for testing in Pujolar et al. 2023 or could be related to temporal shifts in the hybrid zone.

Taken together, our analyses clearly reveal two divergent genetic lineages in US Atlantic *Mnemiopsis*. These results are congruent with evidence of genetic differentiation between Massachusetts and Florida in a previous study (22). However, unlike previous studies, our high-resolution spatial sampling has revealed a clear genetic breakpoint at Roanoke Island, NC. This breakpoint is located where the Gulf Stream leaves the coast and moves offshore into the open Atlantic Ocean. Contrary to expectations for a holopelagic species, we do not find evidence of gene flow between lineages, except at the hybrid zone. Importantly, this hybrid zone is particularly narrow, with admixture observed at Roanoke Island but absent from Piver’s Island, NC located ~200 km away, highlighting that despite their holopelagic nature, clear barriers exist preventing panmixia in *Mnemiopsis*.

### Demographic history of *Mnemiopsis*

Stairway plot (33) analysis shows that the demographic histories of *Mnemiopsis* could be traced back from very recent history (<100 generations/years ago) to approximately 100,000 years ago (Figure 2C). The demographic trajectories of the two lineages were remarkably different with the southern lineage experiencing constant population expansion, especially at about 10,000 years ago. The northern lineage has experienced two population bottlenecks, occurring roughly 10,000 years ago and 2,000–3,000 years ago. More recently, the northern lineage is experiencing a population expansion. Interestingly, the split in the effective population size curves between the two lineages occurred ~10,000 years ago which coincides roughly with the retreat of the Laurentide Glacier (by ~8,000 years ago this glacier was confined mostly to Canada) and the flood that was caused when Lake Agassiz burst and flooded several parts of North America, including the northern Atlantic Ocean (34). This influx of cold, freshwater may have reduced the current in the ocean conveyor belt and resulted in less warm seawater traveling poleward and subsequently causing temperatures to drop in the Northern Hemisphere (34, 35). This population size split may have been a result of a reduction in the Gulf Stream current during this time which could have resulted in *Mnemiopsis* to move northward and begin a population expansion as temperatures slowly rose. Finally, the population size decline around 2,000 – 3,000 years ago in the northern lineage may have been caused by the Little Ice Age which occurred ca. 1300–1850 CE (36, 37).

Our results differ from the pairwise sequentially Markovian coalescent (PSMC) results reported in Jaspers et al (22). Although they also reported population expansions about 50,000 generations ago and a population bottleneck about 1,000–2,000 generations ago, their northern lineage did not show evidence of oscillations in population size. In contrast, ours showed a steady increase in population size beginning at that time. Their southern lineage showed a steady increase in population size from 500,000–50,000 generations ago and then a decrease in population size from then onwards whereas ours has shown a steady increase from 100,000 generations ago to present. The difference in findings can likely be explained by the increased geospatial resolution with our study drawing conclusions from whole-genome level sampling of 5 northern populations and 8 southern populations versus one northern and southern population in the previous study. In addition, while the PSMC method (38) performs well for inferring ancient histories, the Stairway Plot method performs better for inference of more recent population histories and we see the most discordance in our results when looking at more recent population histories (33, 39).

### Structural genomic evidence of speciation

To investigate genomic differences between the two species, we produced two high-quality, near chromosome-level genome assemblies of one Massachusetts *Mnemiopsis* sample and one Florida sample using PacBio Hifi sequencing technology. When assembled, the Florida genome was 206 Mb and half of the genome was contained in contigs 3.6 Mb or larger (N50). There were 174 contigs total and the 100 largest contigs made up 98% of the assembly. The Massachusetts genome was 215 Mb and half of the genome was contained in contigs 2 Mb or larger. There were 207 contigs total and the 100 largest contigs made up 89% of the assembly. We identified 27,570 and 26,046 protein-coding genes for Florida and Massachusetts, respectively and were able to functionally annotate 27,476 genes for the Florida genome. Benchmarking Universal Single-Copy Orthologs (40) analysis revealed that our assemblies exhibit a similar degree of completeness to other ctenophore genomes (Table 2).

**Figure 3.**
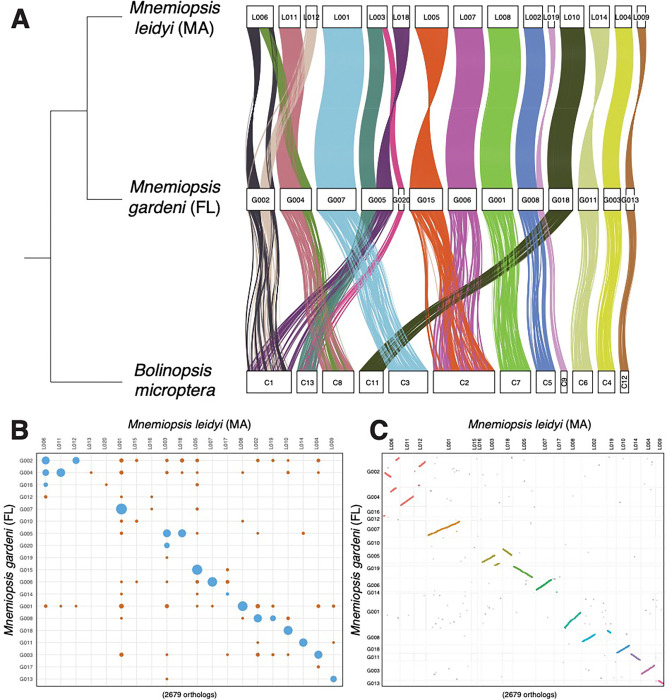
Macrosynteny on a near-chromosome scale between *Mnemiopsis leidyi* (top), *M.* gardeni (middle), and *Bolinopsis microptera* (bottom). **(A)** Chord diagram of macrosynteny between *M. leidyi* (MA), *M. gardeni* (FL), and *B. macropteri*. The coordinates of 12,134 orthologs (based on reciprocal best BLAST hits) are plotted. **(B)** Plot of macrosynteny between *M. leidyi* and *M. gardeni*. **(C)** Oxford dot plot between *M. leidyi* (MA) and *M. gardeni* (FL). Each dot represents an ortholog.

Chromosome-scale linkages of orthologous genes tend to be conserved even over very large time scales (41–45). As such, chromosomal rearrangements are major evolutionary events that can either cause speciation events and/or provide evidence of long periods of isolation between lineages. We conducted macrosynteny analyses to check for evidence of chromosomal rearrangements between the Florida and Massachusetts near-chromosomal genomes. In addition, we compared both genomes to the recently published chromosome-level genome assembly of *Bolinopsis microptera* (44), a lineage that is closely related to *Mnemiopsis* (46). We identified at least one chromosomal rearrangement between the Florida and Massachusetts genomes (Figure 3A–C). The Massachusetts scaffold L006 maps to two Florida scaffolds, G002, which maps completely to Chromosome 1 of *B. microptera*, and G004, which maps completely to chromosome 8 of *B. microptera*. The fact that this linkage group is present in both Florida *Mnemiopsis* and *B. microptera*, suggests that the genomic rearrangement occurred in the Massachusetts lineage. There are potentially other rearrangements involving the Massachusetts scaffolds L012 and L018, but a chromosome-scale assembly would be needed to confirm or refute those possibilities.

### The combined evidence supports two species along the US Atlantic coast

Both the population genomic and structural genomic evidence are consistent with there being one species of *Mnemiopsis* (*Mnemiopsis leidyi*) that inhabits the coastal waters stretching from the Outer Banks region of North Carolina to the northern limits of the native range (i.e., at least as north as Woods Hole, MA). Likewise, there is a second species that stretches from at least the Gulf of Mexico to Piver’s Island, NC.

The position of this break corresponds with the location where the swift-flowing Gulf Stream diverges from the North Carolina coast as it collides with the Labrador current (47). This is a known dispersal barrier, and it is thought that these ocean flows are the source of range limits for many species (48). This seems to serve as the likely biogeographic barrier separating *Mnemiopsis leidyi* (north) from *Mnemiopsis gardeni* (south). During the last glacial maximum, the Gulf Stream followed its present-day course, but its flow was relaxed by at least 10% (49). As the Laurentide glacier receded and led to a habitat hospitable to *Mnemiopsis*, this reduction of flow may have been sufficient to allow a small founder population to overcome this barrier and these individuals may have initiated the present day *Mnemiopsis leidyi* lineage. A small founding population would be consistent with drastic genomic changes like chromosomal rearrangements being tolerated and spreading to fixation.

### Genomic signatures of selection in genes mediating environmental sensing

Due to the substantial divergence between these two species, their geographical distribution that spans a significant environmental gradient, and their success as an invasive species, we investigated signatures of selection and local adaptation between the two lineages using the cross-population composite-likelihood ratio (XP-CLR) test (50). To highlight functional categories of these genes, we conducted gene ontology (GO) analysis (51) of the 2,093 genes that overlap with the genomic windows ranked in the top 10% of XP-CLR values. These genes were significantly enriched (P<=0.05) for GO molecular function categories (Table 3). Interestingly, many of these categories include categories associated with sensory processing, neural activity, and/or diet, for example G protein-coupled receptor signaling pathways (P=0.0004), extracellularly glutamate-gated ion channel activity (P=0.012), Golgi vesicle transport (P=0.0078), neurotransmitter transport (P=0.0321), and chitin metabolic processes (P=0.0043).

**Figure 4.**
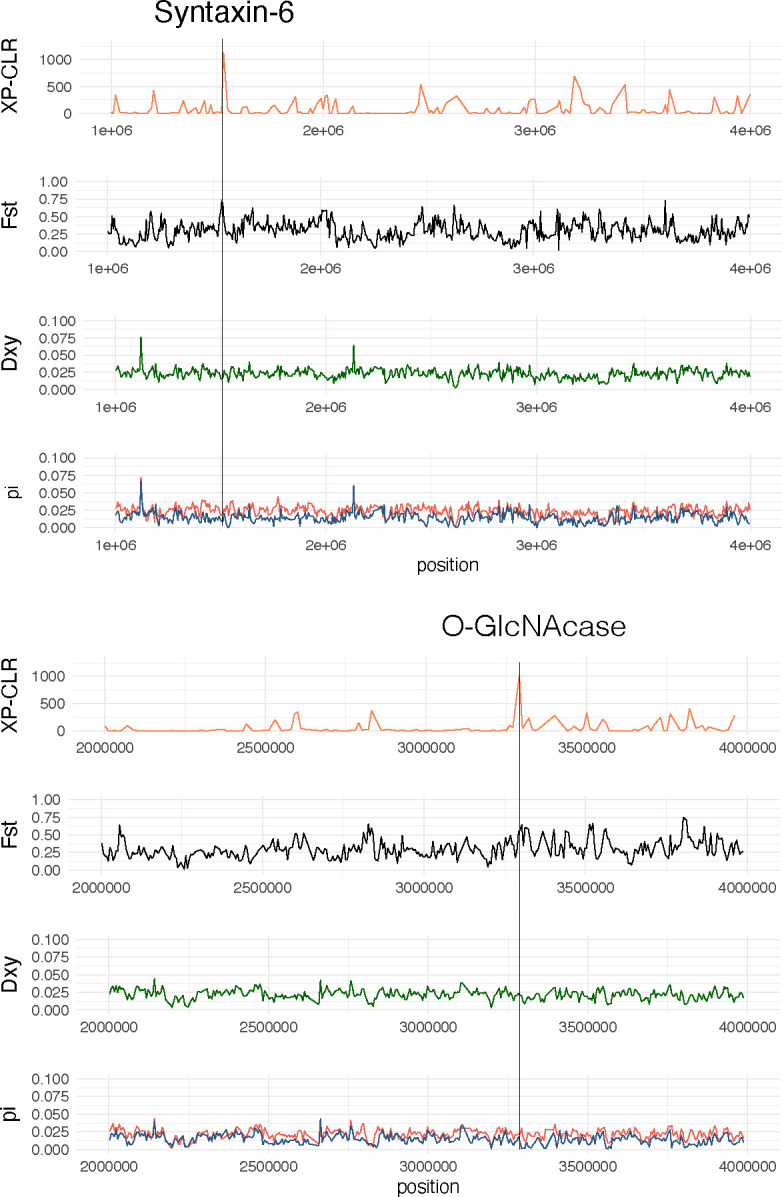
The two genes: syntaxin-6 (top) and O-GlcNAcase (bottom) are under selection. The XP-CLR, F_ST_, D_XY,_ and nucleotide diversity values were calculated with nonoverlapping 10kb windows and step sizes. The location of the two genes is shown with a dotted gray line.

The membrane category, which was significantly enriched (P=0.0122), included several key sensory related and neural genes found in regions with high XP-CLR values such as six TRP channels, four glutamate receptors, the single *Mnemiopsis* voltage-gated calcium channel, one of the two *Mnemiopsis* voltage-gated sodium channels, nine voltage-gated potassium channels, two potassium 2-pore domain channels, two calcium channels, a calcium-activated potassium channel, a voltage-gated chloride channel, and two ASIC channels. The chitin metabolic processes category includes two chitinase genes, which are critical for digesting prey. The membrane category also includes syntaxin-6, which is in the genomic region with the highest fixation index of the XP-CLR analysis (Figure 4). Within the XP-CLR outlier regions, we also observe *O*-GLcNAcase (Figure 4) which is a post-translational modifier of serine and threonine residues on a wide range of proteins (52). This protein has been associated with stress responses ranging from heat shock to oxidative stress. As both syntaxin-6 and *O*-GLcNAcase have been implicated in modulating autophagic flux in response to nutrient status in *Caenorhabditis elegans* (53, 54), we hypothesize that overwintering in northern populations may be a driver of these selective sweeps as organisms are likely nutrient deficient during this time. In addition, our outlier dataset also contains many other genes that are relevant for nutrient sensing and metabolism such as mechanistic target of rapamycin (mTOR), acetyl coenzyme A synthase, malate dehydrogenase, and cytochrome c oxidase subunit 2. Combined, these results suggest that genes involved in sensing a changing environment and adapting to changes in diet were under high positive selection in the northern lineage as it invaded new environments. This finding provides important candidate genes for future functional verification and sheds light on the importance of nutrient sensing and resilience to nutrient deficiency which may make *Mnemiopsis* such a prolific invader.

## Conclusion

Our knowledge of evolution is heavily biased across the tree of life and expansions in taxon sampling often leads to a much richer understanding of how evolutionary forces shape the genomic variation between populations and species. Our findings in the holopelagic ctenophore, *Mnemiopsis* show that despite having life history traits that should promote panmixia (i.e., high fecundity, large population sizes, and high dispersal potential), we instead find evidence of a strong genetic breakpoint that is likely due to barriers created by strong currents off the North Carolina coast. We provide evidence that implicates the retreat of the Laurentide glacier and the subsequent flood of Lake Agassiz in the lineage splitting of the northern and southern species. Finally, we show evidence for selection on environmental sensing genes and genes implicated in surviving stressful conditions, all of which likely contribute to the invasive success of *Mnemiopsis*.

## Materials and Methods

### Sampling

#### Populations.

A total of 118 *Mnemiopsis* samples were collected from thirteen locations along the Atlantic and Gulf coast of the United States. These locations included: Woods Hole (MA), Little Egg Harbor Township (NJ), Berlin (MD), Gloucester Point (VA), Roanoke Island (NC), Pivers Islands (NC), Wrightsville Beach (NC), Charleston (SC), Savannah (GA), Savannah offshore (GA), Flagler Beach (FL), Fort Pierce (FL), and Panacea (FL). Samples were collected between August 2021 and November 2022 from surface waters (<10m) using either a cteno-dipper or a tow net. Samples immediately preserved in ethanol and placed in a −20°C freezer until further processing.

#### Genome.

One *Mnemiopsis* individual was collected from two locations for genome assembly: Woods Hole (MA) and Flagler Beach (FL). These samples were collected in August 2023 from surface waters (<2m) using a cteno-dipper. Samples were preserved in ethanol and placed in a −20°C freezer until further processing.

### DNA Extraction and Sequencing

#### Populations.

High molecular weight DNA was extracted according to the CTAB/Chloroform protocol detailed in Ehrlich et al. 2017 (55). All DNA extractions were visualized on a 1% agarose gel for quality control. One hundred and nineteen individually indexed, paired-end libraries with an approximate insert size of 550 bp were constructed using the NEBNext Ultra II FS DNA Library Preparation kit. Whole-genome sequencing was performed at the University of Florida’s ICBR Bioinformatics Core Facility on a NovaSeq6000.

#### Genome.

High molecular weight DNA was extracted using a modified SDS-based lysis described in Moritz et al (55). DNA extractions were visualized on a 1% agarose gel for quality control and quantified using a Qubit dsDNA High Sensitivity Assay Kit on a Qubit 2.0 Fluorometer. Genome sequencing was performed on a Pacific Biosciences Sequel IIe platform, in the Circular Consensus sequencing (CCS) mode. A total of 17.7 – 20.2 Gb of Hifi reads were generated using CCS for the two individuals. PacBio library preparation and sequencing were performed at NIH Intramural Sequence Center (NISC) in Rockville, MD.

### De novo Genome Assembly

Hifiasm v0.19.8 (56) was used to assemble the two genomes. For the Flagler Beach, FL individual, the <Monospace>--hom-cov</Monospace> flag was set to 90. To reduce the amount of duplication, PurgeHaplotigs v1.1.2 (57) was run with the ‘contigcov’ option for low coverage, low point between the peaks, and high coverage cutoffs set to 10, 70, and 175 respectively. The percent cutoff for identifying a contig as a haplotig was set to 50% and this assembly was subsequently run through PurgeHaplotigs ‘clip’ option that identifies and trims overlapping contig ends. For the Woods Hole, MA individual, the <Monospace>--hom-cov</Monospace> flag was set to 101, the ‘contigcov’ options were set to 25, 70, and 155, and the <Monospace>-j</Monospace> and <Monospace>-s</Monospace> flags were both set to 55. The percent cutoff <Monospace>-a</Monospace> flag was set to 60% and the final assembly was also clipped to remove overlapping contig ends.

### Gene Prediction and Annotation

Genomic repetitive elements were identified with RepeatModeler v1.0.11 (58) to generate a *Mnemiopsis*-specific repeat element library. Repetitive regions were soft masked prior to gene prediction and annotation using RepeatMasker v4.0.8. We aligned previously published bulk RNA-Seq reads from tissues (PRJEB28334) to the above genome assembly using Bowtie2 (59). Aligned RNA-seq data and gene models for *M. leidyi* (ML2.2.aa) were input into Braker v2.1.5 (60). Assembled gene/protein models were functionally annotated using BLASTP v2.9.0+ with three protein databases: UniProt Knowledgebase Swiss-Prot protein models v2021–03, RefSeq invertebrate protein models, and the *M. leidyi* protein models (18). Finally, BUSCO v5 (40) was used to measure the completeness of the genome assembly and protein using the something database.

### MacrosyntR

We analyzed conserved chromosome-scale synteny (macrosynteny) between these new Florida and Massachusetts *Mnemiopsis* assemblies as well as the published *Bolinopsis microptera* genome assembly. To identify conserved genes, we performed diamond BLAST (61) to compute the reciprocal best BLAST hits between the 2013 ML2.2 *Mnemiopsis* gene models and the gene models of each of the new *Mnemiopsis* assemblies and the published *B. microptera* gene models. These reciprocal best BLAST hits were used to identify significantly conserved macrosyntenic blocks in MacrosyntR (version 0.3.3; (62)).

### Data processing and filtering of 118 short-read genomes

Raw sequence data was trimmed using Trimmomatic v0.39 (63) with a minimum phred score of 33. Once the data was filtered and adaptors were removed, the paired sequences were aligned to the Flagler Beach, FL genome using BWA-mem v0.7.12 (38). Sequence alignments were filtered using Samtools v1.9 (64) for a minimum quality score of 30, Sambamba v0.6.6 (65) was used to filter duplicate reads with default parameters, and bamutil v1.0.15 (66) was used to clip overlapping read pairs.

We generated three datasets for our different analyses. For each dataset, we used the Flagler Beach, FL genome as our reference and ancestral genome. *LD-Filtered Dataset.* The first was a highly filtered dataset that was produced by using ANGSD v0.921 (67) to call SNPs and estimate genotype likelihoods using the samtools model (<Monospace>-GL 1</Monospace>). We filtered the SNP data for 1) positions that were present in at least 94 out of 118 samples, 2) a maximum coverage of 2360x per site across all individuals to avoid calling sites in highly repetitive regions, 3) a minimum of 354x coverage per site across all individuals, 4) a minimum base quality score <20, 5) a p-value cut off of 10–6 for calling polymorphic loci and 5) retained only SNPs with a minor global allele frequency >5%. The ANGSD output was then subsampled for one SNP out of every 50 and pairwise linkage disequilibrium (LD) was estimated with ngsLD v1.1.1 (68) on this subsampled dataset assuming a maximum distance of 10Kb between SNPs. Linked sites were pruned with the script ‘prune_graph.pl’ with a maximum distance of 10 Kb between SNPs and a minimum weight of 0.5 (69). The final LD-filtered dataset was used to generate phylogenetic trees, ngsadmix and MDS plots, and calculate F_ST_ measures. *Population-Specific Filtered Dataset.* The second dataset was filtered using the same ANGSD commands as the first dataset (excluding the LD filtering steps) but then underwent a second round of population- specific filtering. Specifically, sampling locations from Woods Hole, MA to Roanoke Island, NC were included in the ‘Northern lineage’ group and sampling locations from Piver’s, NC to Panacea, FL were included in the ‘Southern lineage’ group. These two groups were filtered separately and for the northern lineage, we filtered the dataset for 1) positions that were present in at least 36 out of 45 individuals, 2) a maximum coverage of 900x coverage per site across all individuals, 3) a minimum of 36x coverage per site across all individuals. For the southern lineage we filtered the dataset for 1) positions that were present in at least 58 out of 73 individuals, 2) a maximum coverage of 1,460x coverage per site across all individuals, 3) a minimum of 175x coverage per site across all individuals. We restricted the secondary lineage-specific filtering step to the subset of positions that were retained in the first round of filtering using the -sites option in ANGSD. Filtering in this manner guarantees that sites with an allele that is fixed in one lineage will be retained. The final population-specific filtered dataset was used to generate stairway plots, sliding window F_st_ values, XP-CLR values, and to calculate *f*_3_ statistics. *All-Sites Filtered Dataset.* The third dataset was filtered with the same method as the population-specific dataset, but we kept all positions including non-polymorphic sites. This dataset was used to calculate nucleotide diversity and D_XY_ values.

### Population Structure Analysis

We used the ngsDist option in ngsTools (70) on the LD-Filtered dataset to compute genetic distances between individuals and perform multiple dimensional scaling (MDS). We then looked for signatures of admixture using ngsAdmix (31) with runs ranging from K =2 to K = 7. Weighted pairwise F_ST_s were estimated between the individuals from the North (Woods Hole, MA to Roanoke Island, NC) and the individuals from the South (Pivers Island, NC to Panacea, FL) using ANGSD with the <Monospace>-doSaf 1</Monospace> flag. We then used realSFS to estimate the 2D site frequency spectrum for each population and calculated the average pairwise weighted F_ST_ using ‘realSFS fst.’ Weighted pairwise F_ST_ estimates were also calculated between each sampling location using ANGSD with the same parameters.

NgsDist was used to compute genetic distances from genotype probabilities with *Bolinopsis infundibulum* as the outgroup (SRX14647678). Raw *Bolinopsis* sequences were filtered according to the same protocol as was applied to *Mnemiopsis* and then we ran ANGSD with *Bolinopsis* included, a minor allele frequency filter of 0.05 and a SNP pval filter of 1e-6. This set of filters resulted in 1,302 SNPs that were used to generate the phylogenetic tree based on the nuclear genome. NgsDist was run with 100 bootstrap replicates and a boot block size of 20. Finally, FastME v2.1.6.1 (71) was used to visualize the pairwise genetic distances and RAxML-NG v0.9.0 (72) was used to place bootstrap supports onto the tree. Final figures were generated using the interactive tree of life tool v6.9.1 (73).

Novoplasty v4.3.1 (74) was used to extract the mitochondrial genome from each trimmed sample using the published *Mnemiopsis leidyi* COI sequence (KF435121.1(75)). When this was unsuccessful, we instead used the published *Bolinopsis microptera* (SRX10473759 (46)) whole-genome sequences and ran Novoplasty with the published *B. microptera* COI seed (MW735734.1 (76)) to extract the complete mitochondrial genome. The complete *B. microptera* mitochondrial genome was then used as a seed for the remaining samples that initially failed Novoplasty. Using this approach, we were able to successfully assemble 112 out of 118 *Mnemiopsis* mitochondrial genomes. Next, we used MARS (77) to ensure that each mitochondrial genome began at the same location using the branch and bound method and the block length set to 100. We used MAFFT v7.480 (78) to generate an alignment and IQ-TREE v2.3.2 (79) to generate a phylogenetic tree.

### Demographic Analysis

StairwayPlot v2 (33) was used to reconstruct effective population sizes based on the folded SFSs generated from the All-Sites Filtered dataset. We used a mutation rate of 6.85 × 10^−8^ per base pair per year (22) and a generation time of 1 year (80). A one-year generation time was chosen based on Charlesworth et al 1994 (81) who asserted that generation time can be estimated as the mean age at reproduction for a female. The hermaphroditic *Mnemiopsis* can reproduce within the first month of its lifespan (82) its lifespan is approximately 2 years (80) and there is no information indicating that they ever lose the ability to reproduce. As such a mean age of reproduction was set to 1 year.

### *F*_3_ Statistics

We used *f*_3_ statistics, included as part of the ADMIXTOOLS package v.7.0.2 (32), as a direct test for detecting hybridization. The *f*_3_ statistic estimates whether allele frequency differences between each population combination deviates more than expected because of incomplete lineage sorting, which implicates admixture. If the *f*_3_ statistic is negative, this suggests that admixture has likely occurred. For this analysis, all populations which belonged to the northern mitochondrial lineage were combined into one population and all the populations which belonged to the southern mitochondrial lineage were combined into the second population. These two lineages were tested against Roanoke Island, NC and Gloucester Point, VA.

### Genomic Signatures of Selection

To detect regions of the genome under natural selection between the two lineages, we used XP-CLR (50) which uses the AFS to calculate local deviations between populations. We used a sliding window and step size of 10kb with the maxSNPs flag set to 200. The genomic regions in the top 10% of the XP-CLR values were considered to represent selective sweeps and were associated with overlapping genes using Bedtools v.2.29.0 (83). We conducted a Monte Carlo analysis to identify overrepresented gene ontology terms as was conducted in Babonis et al. 2018 (84).

To further investigate genomic patterns of regions under selection, we generated sliding window scans for F_ST_, D_XY_, and nucleotide diversity values. To generate the sliding window scans, we used the population-specific filtered dataset with the Roanoke Island, NC population removed. We then used realSFS F_ST_ index in ANGSD to compute per-site F_ST_ indexes and realSFS F_ST_ stats2 to perform a window-sliding analysis with the window and step size set to 10 kb to ensure nonoverlapping windows. The All-Sites Filtered dataset, with the Roanoke Island population removed, was used to generate nucleotide diversity and D_XY_ values. For nucleotide diversity calculations, we used realSFS saf2theta in ANGSD to generate folded SFS’s and then generated these values along a sliding window with thetaStat do_stat and the window and step size set to 10 kb. For D_XY_ calculations, we used the CalcDxy.R code by Joshua Penalba then averaged the resulting values over the same 10kb windows as the F_ST_ and nucleotide calculations.

## Figures and Tables

**Table 1.** F_ST_ values across all populations of *Mnemiopsis* collected based on the LD-Filtered SNP dataset. These locations included: Woods Hole (MAW), Little Egg Harbor Township (NJR), Berlin (MDA), Gloucester Point (VAV), Roanoke Island (NCX), Pivers Islands (NCB), Wrightsville Beach (NCW), Charleston (SCS), Savannah (GAS), Savannah offshore (GAB), Flagler Beach (FLM), Fort Pierce (FLF), and Panacea (FLP).

	MAW	NJR	MDA	VAV	NCX	NCB	NCW	SCC	GAS	GAB	FLM	FLF	FLP
MAW	X	X	X	X	X	X	X	X	X	X	X	X	X
NJR	0.039	X	X	X	X	X	X	X	X	X	X	X	X
MDA	0.056	−0.003	X	X	X	X	X	X	X	X	X	X	X
VAV	0.215	0.097	0.075	X	X	X	X	X	X	X	X	X	X
NCX	0.327	0.230	0.210	0.113	X	X	X	X	X	X	X	X	X
NCB	0.400	0.328	0.314	0.262	0.230	X	X	X	X	X	X	X	X
NCW	0.402	0.330	0.317	0.266	0.235	0.001	X	X	X	X	X	X	X
SCC	0.402	0.331	0.318	0.266	0.236	0.0003	0.0002	X	X	X	X	X	X
GAS	0.407	0.335	0.322	0.271	0.240	0.001	0.001	0.001	X	X	X	X	X
GAB	0.402	0.330	0.317	0.265	0.235	−0.0003	0.001	0.00004	−0.00002	X	X	X	X
FLM	0.391	0.318	0.305	0.253	0.223	0.029	0.028	0.027	0.032	0.027	X	X	X
FLF	0.404	0.331	0.318	0.265	0.235	0.061	0.058	0.087	0.064	0.057	0.03	X	X
FLP	0.422	0.349	0.336	0.283	0.254	0.089	0.086	0.087	0.092	0.086	0.059	0.039	X

**Table 2.** Comparison of genome assembly benchmarks between the *Mnemiopsis leidyi* published genome (18) and the two new genome assemblies.

	*Mnemiopsis leidyi* 2013	*Mnemiopsis leidyi* 2024	*Mnemiopsis gardeni* 2024
Assembly size	155,865,547	215,818,400	206,611,897
No. of scaffolds	NA	5,100	NA
No. of contigs	NA	207	174
Max length (bases)		6,852,836	9,685,951
N50	187,314 (scaff N50)	2,034,471 (contig N50)	3,675,794 (contig N50)
BUSCO Complete	204 (80%)	211 (83%)	214 (84%)
BUSCO Complete + Partial	233 (91%)	231 (91 %)	235 (91 %)
BUSCO missing	22 (8.6%)	24 (9%)	20 (8%)
BUSCO duplicated	1.47	1.9	3.74

**Table 3.** Gene Ontology (GO) term analysis reveals enrichment in relevant biological processes.

GO TERM	P-Value	Description
GO:0005515	0	protein binding
GO:0003723	0.0002	RNA binding
GO:0004553	0.0002	hydrolase activity
GO:0007186	0.0004	G protein-coupled receptor signaling pathway
GO:0005215	0.001	transporter activity
GO:0005524	0.0014	ATP binding
GO:0006468	0.0014	protein phosphorylation
GO:0005488	0.0014	binding
GO:0004930	0.0016	G protein-coupled receptor activity
GO:0004672	0.0016	protein kinase activity
GO:0007018	0.0039	microtubule-based movement
GO:0006030	0.0043	chitin metabolic process
GO:0003755	0.0044	peptidyl-prolyl cis-trans isomerase activity
GO:0000413	0.0044	protein peptidyl-prolyl isomerization
GO:0007165	0.0046	signal transduction
GO:0006366	0.0064	transcription by RNA polymerase II
GO:0030117	0.0074	membrane coat
GO:0048193	0.0078	Golgi vesicle transport
GO:0003676	0.0082	nucleic acid binding
GO:0045454	0.0083	cell redox homeostasis
GO:0008081	0.0098	phosphoric diester hydrolase activity
GO:0005234	0.012	extracellularly glutamate-gated ion channel activity
GO:0016020	0.0122	membrane
GO:0055085	0.0128	transmembrane transport
GO:0005868	0.0143	cytoplasmic dynein complex
GO:0008061	0.0147	chitin binding
GO:0009055	0.0156	electron transfer activity
GO:0006452	0.0157	translational frameshifting
GO:0045905	0.0157	positive regulation of translational termination
GO:0045901	0.0157	positive regulation of translational elongation
GO:0043022	0.0159	ribosome binding
GO:0016462	0.0164	pyrophosphatase activity
GO:0016021	0.0174	
GO:0005216	0.0176	monoatomic ion channel activity
GO:0005096	0.019	GTPase activator activity
GO:0010181	0.0192	FMN binding
GO:0005978	0.0196	glycogen biosynthetic process
GO:0005622	0.0198	intracellular anatomical structure
GO:0006811	0.0223	monoatomic ion transport
GO:0015035	0.0231	protein-disulfide reductase activity
GO:0004970	0.0235	glutamate-gated receptor activity
GO:0008017	0.0243	microtubule binding
GO:0005576	0.0256	extracellular region
GO:0003777	0.026	microtubule motor activity
GO:0004252	0.0268	serine-type endopeptidase activity
GO:0004177	0.0282	aminopeptidase activity
GO:0051056	0.0289	regulation of small GTPase-mediated signal transduction
GO:0046933	0.0292	proton-transporting ATP synthase activity
GO:0006820	0.0311	monoatomic anion transport
GO:0006836	0.0321	neurotransmitter transport
GO:0005328	0.0326	neurotransmitter:sodium symporter activity
GO:0006810	0.033	transport
GO:0016651	0.033	oxidoreductase activity
GO:0019001	0.0395	guanyl nucleotide binding
GO:0031683	0.0395	G-protein beta/gamma-subunit complex binding
GO:0005452	0.0398	solute:inorganic anion antiporter activity
GO:0004674	0.0412	protein serine/threonine kinase activity
GO:0006270	0.0423	DNA replication initiation
GO:0008603	0.0426	cAMP-dependent protein kinase regulator activity
GO:0005952	0.0426	cAMP-dependent protein kinase complex
GO:0006383	0.0426	transcription by RNA polymerase III
GO:0001932	0.0426	regulation of protein phosphorylation
GO:0051287	0.0429	NAD binding
GO:0005815	0.0442	microtubule organizing center
GO:0046872	0.0444	metal ion binding
GO:0005975	0.0449	carbohydrate metabolic process
GO:0043130	0.0452	ubiquitin binding
GO:0046856	0.0455	phosphatidylinositol dephosphorylation
GO:0051920	0.0461	peroxiredoxin activity
